# Type A aortic dissection after ‘zone 0’ thoracic endovascular aortic repair for type 1 hybrid aortic arch replacement of arch aneurysm

**DOI:** 10.1093/jscr/rjaa508

**Published:** 2020-12-18

**Authors:** Sung Joon Han, Man-Shik Shim, Woo Sik Han, Hyun Jin Cho, Min-Woong Kang, Shin Kwang Kang, Jae Hyeon Yu, Myung Hoon Na

**Affiliations:** Department of Thoracic and Cardiovascular Surgery, School of Medicine, Chungnam National University, Daejeon, Republic of Korea; Department of Thoracic and Cardiovascular Surgery, School of Medicine, Chungnam National University, Daejeon, Republic of Korea; Department of Thoracic and Cardiovascular Surgery, School of Medicine, Chungnam National University, Daejeon, Republic of Korea; Department of Thoracic and Cardiovascular Surgery, School of Medicine, Chungnam National University, Daejeon, Republic of Korea; Department of Thoracic and Cardiovascular Surgery, School of Medicine, Chungnam National University, Daejeon, Republic of Korea; Department of Thoracic and Cardiovascular Surgery, School of Medicine, Chungnam National University, Daejeon, Republic of Korea; Department of Thoracic and Cardiovascular Surgery, School of Medicine, Chungnam National University, Daejeon, Republic of Korea; Department of Thoracic and Cardiovascular Surgery, School of Medicine, Chungnam National University, Daejeon, Republic of Korea

## Abstract

The recent rise in minimally invasive cardiovascular procedures is being accompanied by an increase in related complications. We report on an acute type A aortic dissection performed in an 82-year-old man 1 week after staged ‘zone 0’ hybrid thoracic endovascular aortic repair (TEVAR). Previously, the patient had undergone type I hybrid arch debranching and staged ‘zone 0’ TEVAR for an aortic arch aneurysm. ‘Zone 0’ TEVAR after type I hybrid debranching might increase the risk for aortic injury on the residual native aorta and should, therefore, be closely followed up to enable the early diagnosis of complications.

## INTRODUCTION

### Case report

**Figure 1:**
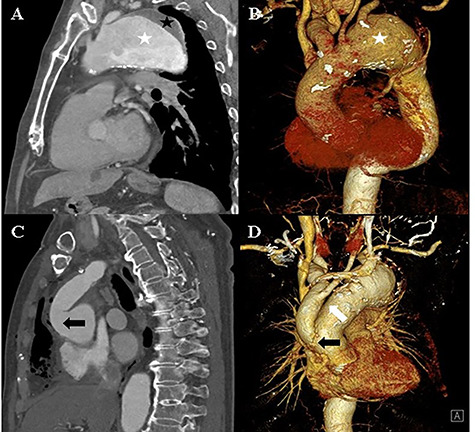
**A** and **B:** Computed tomography of the chest shows an aortic aneurysm (white asterisk) with a mural thrombus (black asterisk) at the distal arch. **C** and **D:** Post-debranching operative computed tomography of the chest shows the graft, which is directly sutured onto the ascending aorta (black arrow), and the graft used from the innominate artery to the left common carotid artery (white arrow).

An 82-year-old man with a history of atypical chest pain was diagnosed with an aortic aneurysm with an eccentric mural thrombus at the distal arch. The thrombus had a maximum diameter of 70 mm starting directly below the ostia of the common carotid artery (Fig. 1). There were no abnormal findings, except for mild aortic regurgitation on preoperative echocardiography. We planned to perform a hybrid thoracic endovascular aortic repair and decided to carry out the debranching operation first. A type I hybrid arch debranching operation was performed; an 18-mm Gore-Tex graft was used from the ascending aorta to the innominate artery, and another 8-mm Gore-Tex graft was used from the innominate artery to the left common carotid artery in Spielvogel fashion (Fig. 1). There were no post-operative complications.

**Figure 2:**
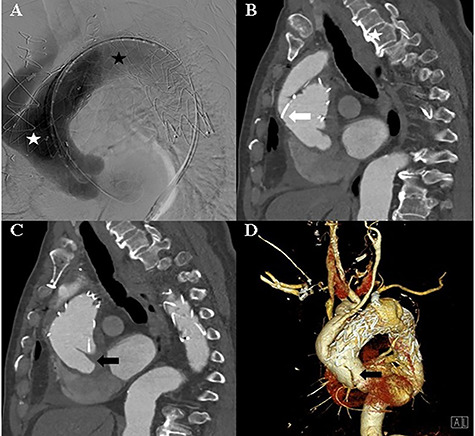
**A:** Aortic angiography during TEVAR shows the thoracic graft implanted in the ascending thoracic aorta and aortic arch (white asterisk) and the thoracic graft implanted in the aortic arch and descending thoracic aorta (black asterisk). **B:** Computed tomography of the post-TEVAR shows the placed thoracic graft (white arrow). **C** and **D:** Computed tomography of the post-TEVAR shows the intimal dissecting flap at the proximal ascending aorta.

The TEVAR was performed 14 days after the debranching surgery. Under local anaesthesia, a Safari wire was placed in the left ventricle cavity. A Medtronic Valiant Thoracic Stent Graft (46 mm × 46 mm × 200 mm) was implanted in the ascending thoracic aorta and aortic arch, and a second Medtronic Valiant Thoracic Stent Graft (42 mm × 42 mm × 200 mm) was implanted in the aortic arch and descending thoracic aorta. The left subclavian artery was plugged with an Amplatzer Vascular Plug (16 mm) (Fig. 2).

**Figure 3:**
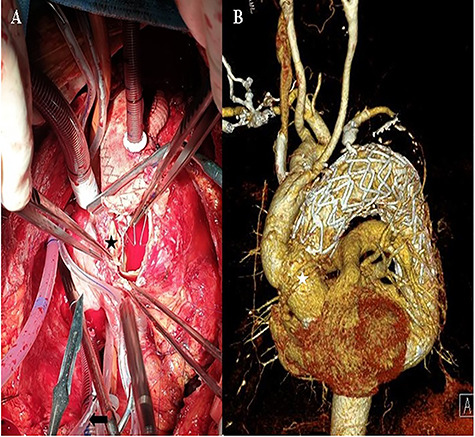
**A:** Intraoperative view of the transected ascending aorta due to the thoracic stent graft (black asterisk). **B:** The post-operation computed tomography image shows the replaced ascending aorta (white asterisk).

The patient remained asymptomatic with stable vital signs. A scheduled computed tomography was performed after 1 week. Computed tomography revealed a newly localized intimal dissecting flap at the proximal ascending aorta, which was located proximally, extending to the ostia of the right coronary artery. The patient underwent an emergency open repair operation. Circulatory arrest was established at a rectal temperature of 20°C, and cerebral perfusion was accomplished through the 18-mm graft cannula and left femoral artery cannulation. During gross examination, the 18-mm graft was seen detached from the ascending aorta, which was opened longitudinally, and an intimal tearing site was identified (Fig. 3). The dissection occurred transversely at the proximal stented site and was extended up to the sinotubular junction around the left coronary sinus and near the right coronary orifice in the right coronary and non-coronary sinuses. A Gelweave 28-mm straight graft was used, which was connected to the proximal aorta and reinforced with the double sandwich technique (Fig. 3). The distal anastomosis was performed, including a proximal resected valiant stent graft and the patient’s native aorta with a Teflon felt by sandwich technique. After the operation, the patient remained asymptomatic.

## DISCUSSION

The recent increase in minimally invasive cardiovascular procedures is accompanied by an increase in related complications, such as access route injury or catheter related dissection (incidence of 1.3–6.8% in previous studies) [1, 2]. Even though the complication rate is relatively low compared with open surgical repair, the mortality rate is high when complications arise [3].

Type A aortic dissections, which are some of the most catastrophic complications, can be caused by retrograde blood flow, injury to the aortic wall or the stent itself [4]. In our case, we cannot exclude the possibility that the dissection was produced by the re-coiling force of the stent itself. Several studies report possible mechanisms related to stent grafts, which seem to be associated with the proximal landing zone. In other words, the incidence of aortic dissection is significantly higher when ‘zone 0’ TEVAR is performed, rather than ‘zone 3’ or ‘zone 4’ TEVAR [5]. According to a recent systematic review study based on a meta-analysis, the spring-back force, which can be described as the inherent force of the spring to return to its original straight form when passively curved, may be the cause. Otherwise, the ‘Windkessel effect’, caused by the dynamic movement of the aorta during the cardiac cycle, may lead to dissection [6].

Type A dissections due to stents often require open surgical repair and result in poor prognoses. Hence, to avoid complications, extremely careful manipulation of the wire, graft or delivery system is mandatory when performing TEVAR, especially ‘zone 0’ TEVAR. A close follow-up is vital to detect complications that can develop either during the procedure or even weeks or months later.

## CONFLICT OF INTEREST STATEMENT

None declared.

## References

[1] Duran C, Naoum JJ, Smolock CJ, Bavare CS, Patel MS, Anaya-Ayala JE, et al. A longitudinal view of improved management strategies and outcomes after iatrogenic iliac artery rupture during endovascular aneurysm repair. Ann Vasc Surg 2013;27:1–7. 22981018 10.1016/j.avsg.2012.04.017

[2] Won JY, Lee DY Problems encountered during and after stent-graft treatment of aortic dissection In: Rousseau H, Verhoye JP, Heautot JF (eds). Thoracic Aortic Diseases. Berlin, Heidelberg: Springer, 2006, 209–22

[3] Eggebrecht H, Thompson M, Rousseau H, Czerny M, Lönn L, Mehta RH, et al. Retrograde ascending aortic dissection during or after thoracic aortic stent graft placement: insight from the European registry on endovascular aortic repair complications. Circulation 2009;120:S276–81. 19752379 10.1161/CIRCULATIONAHA.108.835926

[4] Higashigawa T, Kato N, Chino S, Hashimoto T, Shimpo H, Tokui T, et al. Type a aortic dissection after thoracic endovascular aortic repair. Ann Thorac Surg 2016;102:1536–42. 27316317 10.1016/j.athoracsur.2016.04.024

[5] Williams JB, Andersen ND, Bhattacharya SD, Scheer E, Piccini JP, McCann RL, et al. Retrograde ascending aortic dissection as an early complication of thoracic endovascular aortic repair. J Vasc Surg 2012;55:1255–62. 22265798 10.1016/j.jvs.2011.11.063 PMC3699184

[6] Chen Y, Zhang S, Liu L, Lu Q, Zhang T, Jing Z Retrograde type a aortic dissection after thoracic endovascular aortic repair: a systematic review and meta-analysis. J Am Heart Assoc 2017;6:e004649. 28939705 10.1161/JAHA.116.004649 PMC5634245

